# Identification of Alternative Splicing Events Associated with Paratuberculosis in Dairy Cattle Using Multi-Tissue RNA Sequencing Data

**DOI:** 10.3390/genes13030497

**Published:** 2022-03-11

**Authors:** Houcheng Li, Jinfeng Huang, Junnan Zhang, Yahui Gao, Bo Han, Dongxiao Sun

**Affiliations:** National Engineering Laboratory of Animal Breeding, Department of Animal Genetics and Breeding, College of Animal Science and Technology, China Agricultural University, Beijing 100193, China; sanderslhc@126.com (H.L.); huangjinfeng1003@126.com (J.H.); cauzhangjn@163.com (J.Z.); gyhalvin@gmail.com (Y.G.); hanbo_98@126.com (B.H.)

**Keywords:** paratuberculosis, RNA-seq, differentially expressed gene, alternative splicing, dairy cattle

## Abstract

Paratuberculosis is a major endemic disease caused by *Mycobacterium avium subspecies paratuberculosis* (MAP) infection and leads to huge economic loss in the dairy sector worldwide. Alternative splicing (AS) events, playing indispensable regulatory roles in many protein functions and biological pathways, are shown to be associated with complex traits and diseases. In this study, by integrating the RNA sequencing (RNA-seq) data of 24 samples from three tissues (peripheral blood, jejunum and salivary gland) of Holstein cows, we obtained 2,706,541,696 uniquely mapped reads in total that represented 12,870 expressed genes, and detected 4285 differentially expressed genes (DEGs) between MAP-infected and healthy cows (*p* < 0.05). Of them, 92 differentially expressed splicing factors (DESFs) were included. Further, 119, 150 and 68 differential alternative splicing (DAS) events between MAP-infected and healthy cows were identified in peripheral blood, jejunum and salivary glands, respectively. Of note, six DAS events were highly and significantly correlated with the DESFs (*R*^2^ > 0.9; *p* < 0.01), and their corresponding genes (*COPI coat complex subunit gamma 2* *gene* (*COPG2*), *kinesin family member 2C gene* (*KIF2C*), *exocyst complex component 7* (*EXOC7*), *Rab9 effector protein with kelch motifs gene* (*RABEPK*), *deoxyribonuclease 1 gene* (*DNASE1*) and *early endosome antigen 1* *gene* (*EEA1*)) were significantly enriched in immune response such as vesicle-mediated transport, regulation of acute inflammatory response and tuberculosis through gene ontology (GO) and KEGG analysis. KS test showed that the DAS events in the *EXOC7* and *KIF2C* genes indeed displayed differences between MAP-infected cows and healthy cows. The DAS in *EXOC7* might produce a new protein sequence with lack of 23 amino acids, and the DAS in *KIF2C* induced a stop codon of premature occurrence and resulted in a lack of functional domain. In summary, this study identified the DAS events and corresponding genes related to MAP-infection base on the RNA-seq data from multiple tissues of Holstein cows, providing novel insights into the regulatory mechanisms underpinning paratuberculosis in dairy cattle.

## 1. Background

Paratuberculosis is a chronic and gastrointestinal infectious disease in livestock such as cattle and sheep, which is caused by *Mycobacterium avium subspecies paratuberculosis* (MAP) [1,2]. Infected animals usually have a long incubation period of 6–12 months and develop clinical signs such as diarrhea and dehydration. MAP infections have already caused serious economic losses in dairy farms due to decreased milk production and increased management costs [3,4]. Through traditional farm management, paratuberculosis is difficult to prevent and control because of its long incubation periods and convenient transmissible ways of pathogen such as feces and infected milk. In 2007, Gonda et al. first detected a quantitative trait locus (QTL) associated with susceptibility to MAP infection on BTA20 in US Holsteins [5]. Several QTLs for MAP resistance or susceptibility were identified on chromosomes 7, 16 and 22 with genome-wide association studies (GWASs) in Holstein cattle [6,7]. Gao et al. identified 30 immune-related genes associated with paratuberculosis by performing GWAS [8] and RNA sequencing (RNA-seq) in jejunum tissue in Chinese Holsteins, such as *NOD2*, *SLC11A1*, *TLR*, *SPI10*, *IL10RA*, *BolFNG* and *PGLYRP1* [9].

Alternative splicing (AS) means different combinations between exons and introns in mRNA processing. In the process of pre-mRNA maturation, AS events of genes may generate numerous mature mRNA isoforms in higher eukaryotes [10], which means that a limited number of genes are able to generate vast numbers of proteins via AS events of eukaryotic transcripts in cells, which has notable physiological functions in the different developmental processes [11]. AS events might be enriched for specific pathways and regulate the physiological function in different tissues, such as AS events in brain were enriched for endocytosis and vesicle mediated transport, in heart were enriched for development of cardiomyocytes and ion channels, in liver were enriched for Actin-based processes and protein transport [12]. Some AS events can result in serious changes in mRNA or protein structures such as the domain change and intronic polyadenylation, which are related to diseases. In humans, it has been shown that 370 kinds of diseases are associated with the disruption of AS events and 2337 splicing mutations are linked to common diseases such as skipped exon 10 of *microtubule associated protein tau gene* (*MAPT*) and Alzheimer’s Disease, skipped exon 7 of *fused in sarcoma/translocated in liposarcoma RNA binding protein gene* (*FUS*) and Amyotrophic Lateral Sclerosis, and skipped exon 2 of *cytotoxic T-lymphocyte associated protein 4 gene* (*CTLA4*) and some autoimmune diseases [13,14,15,16,17]. In dairy cattle, 3.66% and 5.4% novel transcripts produced by specific AS events were identified in the mammary tissues from healthy and mastitic cows, respectively, which were related to immune defense and inflammation responses and harbored the known QTL for clinical mastitis [18]. Yang et al. revealed a novel transcript with a deletion of 112 bp of exon 2 in *C-C motif chemokine ligand 5 gene* (*CCL5*) had significant lower frequency in mastitic cows compared with healthy ones [19]. By using isolated ileum segments, Liang et al. identified the expression of exon1 of *monocyte to macrophage differentiation associated gene* (*MMD*) decreased and the expression of intron4 of *adenosine deaminase associated gene* (*ADA*) increased significantly in MAP-infected cows [20].

So far, studies on regulatory mechanism of AS events on susceptibility or resistant to MAP, e.g., which AS event and its corresponding gene play critical roles in MAP infection are still insufficient. In the present study, by integrating our 6 RNA-seq data sets of jejunum from the healthy and clinical Holstein cow of paratuberculosis [8,9], and 18 RNA-seq data sets from Holstein cows before and after MAP-infection from the European Bioinformatics Institute (EMBL-EBI) database (https://www.ebi.ac.uk/ accessed on 20 April 2021) [21], we detected the AS events and corresponding genes and analyzed their potential regulatory roles in paratuberculosis.

## 2. Materials and Methods

### 2.1. RNA Sequencing Data

The jejunum samples for RNA-seq used in this study have been detailed as described in our previous study [9]. Briefly, 6 individuals were selected from 8214 Chinese Holstein cows in Beijing Sanyuan Dairy Farm Center based on serum ELISA detection and stool real-time PCR results [22], i.e., 3 healthy (Non-Positive, NP) and 3 clinical (Double Positive, DP) cows of paratuberculosis. Total RNA of the 6 jejunum samples were sequenced with Illumina Hiseq 2500 technology platform in Novogene (Beijing, China). Consequently, 12 compressed FASTQ files of 112 Gb with around 292,907,510 paired-end reads of 150 bp were obtained. The dataset is available in the NCBI BioProject database under the accession number PRJNA756737.

In addition, 18 paired-end RNA-seq data sets of Holstein cows before and after MAP-infection in the EMBL-EBI database (https://www.ebi.ac.uk/ accessed on 20 April 2021) were downloaded, including 36 compressed FASTQ files of 260 Gb. Of these, there are 14 sequencing samples from salivary gland with around 811,607,061 reads and 4 from peripheral blood with 90,366,248 reads [23,24]. The details of the data sets are summarized in Table 1.

### 2.2. Quality Control of Reads and Reads Alignment to the Bovine Reference Genome

The raw data (reads) were filtered with Trimmatic-0.38 software [25], resulting in high quality clean reads as follows: deletion of reads with linkers; deletion of reads with N (N means that the base information could not be determined) content greater than 10%; deletion of low-quality reads (reads with base quality less than 3 or average base quality less than 15). The clean reads were used for the following analysis.

We downloaded the bovine reference genome ARS-UCD1.2 [26] and corresponding annotation files from Ensembl genome browser (https://www.ensembl.org accessed on 2 May 2021) [27], and employed STAR v2.5.3 [28] to build an index of reference genome for the alignment.

### 2.3. Identification of Differentially Expressed Genes

Read counts for each of the 24,616 annotated Ensembl genes (specified as from transcription start site (TSS) to transcription end site (TES)) were calculated using FeatureCountsv1.5.2 [29] based on sorted bam files ordered from the alignment. The raw read counts were processed using the EstimateSizeFactors function in the DEseq2 R package (1.26.0), corrected for expression outliers, and DEseq2 normalized read counts were obtained by calculating the median of the ratios of read counts for each gene to the geometric mean of all cows as a scaling factor for a given sample [30]. 

Gene expression was first quantified using normalized counts. Next, differential expression analysis was performed by the DEseq2 R package to identify differentially expressed genes (DEGs) between MAP-infected and healthy samples in each tissue [30]. Finally, the DEGs in each comparison were obtained (*p* value < 0.05). Gene lists were functionally annotated using the metascape website (https://metascape.org/ accessed on 18 June 2021) and clusterprofiler (R package) for functional enrichment analysis based on Gene Ontology and KEGG databases [31,32].

### 2.4. Identification and Screening of Differential Alternative Splicing (DAS) Events

rMATs v4.1.0 [33] and leafcutter v0.2.9 [34] were used to identify AS events and quantify their frequency as the percent spliced in (PSI) based on the sorted bam files. rMATs utilizes proportions of exon-exon junction reads to calculate PSI values, whereas a different clustering of introns caused by AS events is the main focus of leafcutter. In addition, as to rMATs, five possible different AS events can be considered, including exon skipping (ES), intron retention (IR), alternative 3′ splicing site (A3SS), alternative 5′ splicing site (A5SS), and mutually exclusive exons (MXE). 

We compared AS events between MAP-infected cows and healthy cows in different tissues, and significantly differential AS (DAS) event was assessed with a ΔPSI > 10% and FDR < 0.05 which means this AS event has a significant difference of over 10% between the two groups [35]. Furthermore, based on two groups of DAS events identified by rMATs and leafcutter, respectively, the intersection of them and corresponding genes in each tissue were screened for the following analysis.

### 2.5. Correlation Analysis between Splicing Factors and AS Events

We downloaded 317 splicing factors (SFs) from the SpliceAid-F database (http://srv00.recas.ba.infn.it/SpliceAidF/ accessed on 8 September 2021) [36,37]. Based on the results of differential expression analysis, differential expressed SFs (DESFs) were selected in the 3 tissues, and expression matrix of them was created to perform correlation analysis with PSI values of screened DAS events in each tissue, respectively. As the results, DESFs and DAS events with high and significant correlation coefficients (*R*^2^ > 0.9 and *p* < 0.01) were selected. Cytoscape v3.5.1 software [38,39] was used to visualize the SF–AS interaction network.

### 2.6. Statistical Analysis and Protein Structure Prediction

KS test was used to verify the significances of differences in each selected DAS event between MAP-infected and healthy cows [40]. To avoid false positives, FDR correction was performed and a corrected FDR < 0.05 was considered to be statistically significant. All statistical boxplots were generated by using R v3.6.0. Based on the NCBI database (https://www.ncbi.nlm.nih.gov/ accessed on 12 October 2021) and SMART website (http://smart.embl-heidelberg.de/ accessed on 12 October 2021), the effects of DAS events on protein sequences and domains were identified [41,42]. 

## 3. Results

### 3.1. Data Summary

A total of 24 RNA-seq samples from three tissues (peripheral blood, jejunum and salivary gland) were processed, including 2,706,541,696 clean reads after the quality control. Alignment of the sequencing reads against the bovine genome ARS-UCD1.2 obtained 81.35–93.13% of uniquely aligned reads across all samples. Consequently, 12,870 genes that were expressed in at least one samples were detected (DEseq2 normalized count > 1).

### 3.2. Differentially Expressed Genes across Tissues

**Figure 1:**
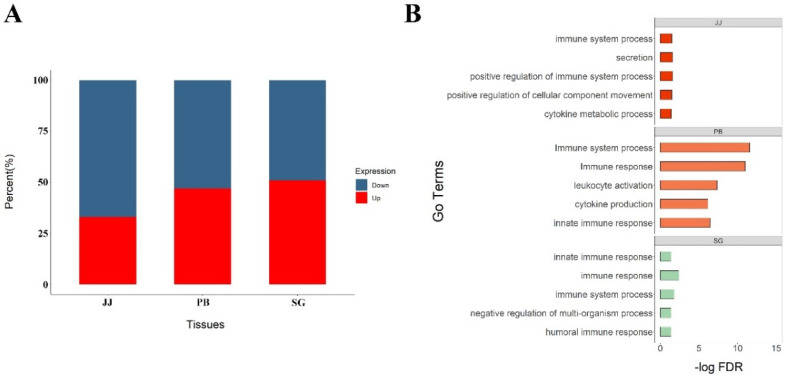
Results of differential expressed analysis. (**A**) The percentage of up–expressed and down–expressed genes of three tissues in infected cows, red parts mean the percent of up–expressed genes, and blue parts mean down–expressed genes; (**B**) Pathway enrichment of differential expressed genes in different tissues.

A total of 4285 DEGs across 3 tissues between MAP-infected and healthy cows (*p* < 0.05) was detected, including 2928, 668 and 1574 DEGs in peripheral blood, jejunum and salivary gland, respectively. Among the case groups of three tissues, the percentage of upregulated genes was significantly higher than that of downregulated genes in jejunum, suggesting there was a global promotion in physiological functions, whereas the percentages of upregulated and downregulated genes were nearly equal in other two tissues, comparing with control groups (Figure 1A). 

Through GO and KEGG analysis, we found the DEGs in jejunum were significantly enriched in biological processes and pathways related to adaptive immune response, including response to cytokine, cytokine production and Th1 and Th2 cell differentiation, whereas the DEGs in salivary gland were enriched in innate immune response and defense response. Additionally, the DEGs in peripheral blood were enriched in both innate immune responses and adaptive immune response (FDR < 0.05). Detailed information about the enriched GO terms and KEGG pathways was shown in Figure 1B.

### 3.3. Differential AS Events

**Figure 2:**
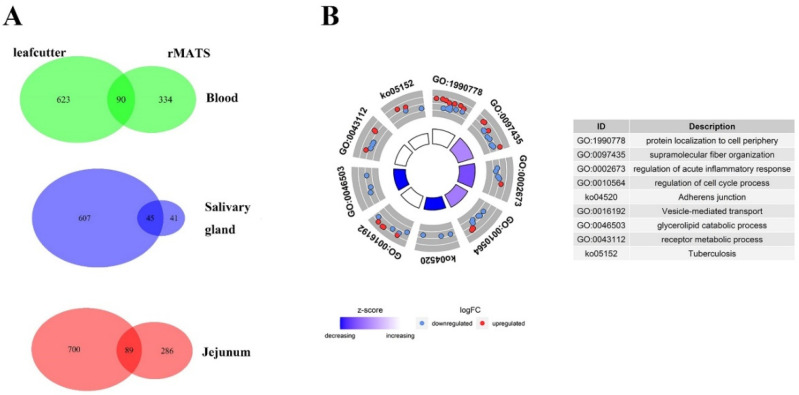
Results of screened DAS events. (**A**) Numbers of screened DAS events. Three Venn diagrams on the left mean numbers of DAS events detected by each software and their overlapped results in different tissues. The one on the right is the intersection of the three tissues. (**B**) Circle figure of pathways enriched results of genes related to screened DAS events. Log2FoldChange values used to calculate Z-score were replaced by ΔPSI values of DAS events.

By using leafcutter, we identified 1242 (713 genes), 1401 (789 genes) and 1168 (652 genes) significantly differential AS (DAS) events between MAP-infected and healthy cows (FDR < 0.05 and ΔPSI > 0.1) in peripheral blood, jejunum and salivary gland, respectively. In addition, 511 (424 genes), 450 (375 genes) and 99 (86 genes) DAS events were identified by rMATs in peripheral blood, jejunum and salivary gland, respectively. To avoid false positives efficiently and access higher reliability, 119 DAS events (90 genes) in peripheral blood, 150 (89 genes) in jejunum and 68 (45 genes) in salivary gland identified by two methods simultaneously were used for further analysis (Figure 2A). The DAS events identified in both software were listed in Supplementary Table S1.

After the deduplication, 224 corresponding genes were used for functional enrichment analysis, and the top 5 enriched pathways of *p*-value were supramolecular fiber organization (*p* = 4.38 × 10^−4^), regulation of acute inflammatory response (*p* = 4.76 × 10^−4^), protein localization to cell periphery (*p* = 6.46 × 10^−4^), regulation of cell cycle process (8.75 × 10^−4^) and vesicle-mediated transport (*p* = 1.32 × 10^−3^). Among these, 14 genes (*CD3G*, *SCARB1*, *MYO5A*, *RABEPK*, *KIF2C*, *EXOC7*, *COPG2*, *REPS1*, *NFYC*, *SYK*, *EEA1*, *CARD9*, *DNASE1*, *TAC1* and *RHBDD3*) were significantly enriched for immune related pathways, including vesicle-mediated transport (*p* = 1.32 × 10^−3^), regulation of acute inflammatory response (*p* = 4.76 × 10^−4^) and tuberculosis (*p* = 2.53 × 10^−3^) (Figure 2B). 

### 3.4. Correlations between Splicing Factors and DAS Events

**Figure 3:**
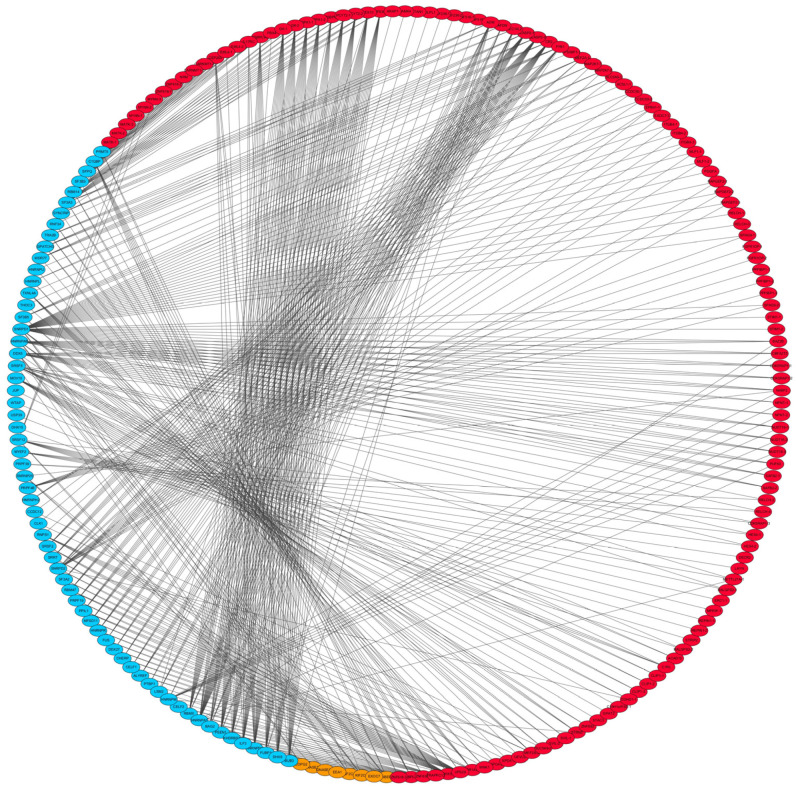
SF−AS interaction network. Gene marked in red or orange means the one with screened DAS event and the suffix such as “−1” or “−2” means each AS event in this gene. Gene marked in blue means the SF. In addition, orange represents the gene enriched for immune system related pathways.

Based on the 4285 DEGs and 317 downloaded splicing factors (SFs), 57, 14 and 21 differential expressed splicing factors (DESFs) were observed in peripheral blood, jejunum and salivary glands, respectively. Results of correlation analysis between DESF and DAS were listed in Supplementary Table S2. Of note, there were high and significant correlations between 62 DESFs (12 in jejunum and 50 in peripheral blood) and 112 DASs (64 in jejunum and 48 in peripheral blood) (*R*^2^ > 0.9; *p* < 0.01). Of these DASs, 6 corresponding genes (*COPG2*, *KIF2C*, *EXOC7*, *RABEPK*, *DNASE1* and *EEA1*) were enriched for vesicle-mediated transport (*p* = 1.32 × 10^−3^), regulation of acute inflammatory response (*p* = 4.76 × 10^−4^) and tuberculosis (*p* = 2.53 × 10^−3^) pathways, suggesting potential relations to the susceptibility to MAP (Figure 3).

### 3.5. Changes of Protein Structure

**Figure 4:**
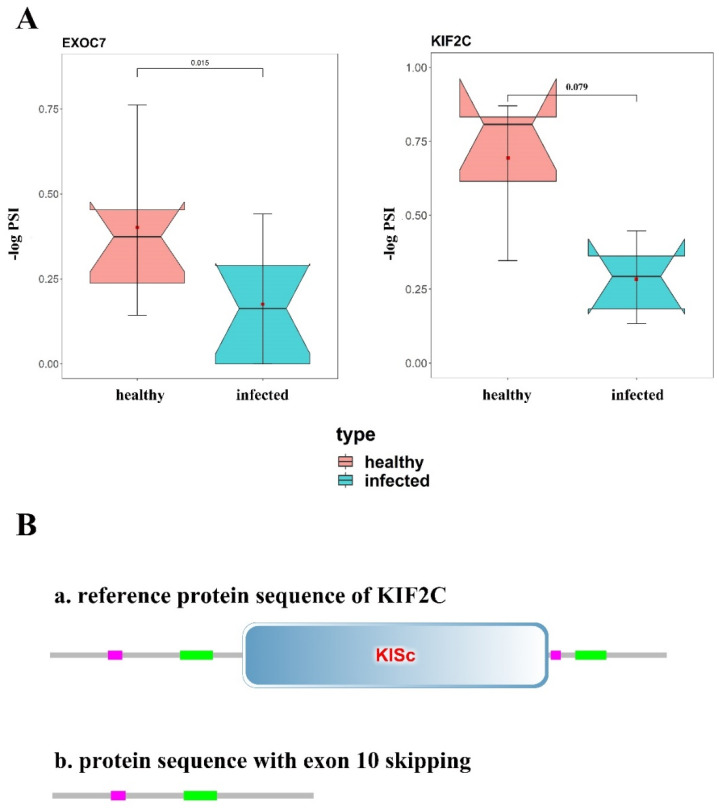
KS−test of candidate DAS events and protein structure prediction of corresponding genes. (**A**) KS test of DAS events in EXOC7 and KIF2C. Dots in red part mean PSI values of DAS event in healthy cows, and blue part mean that in infected cows. (**B**) Protein structure of KIF2C before and after exon 10 skipping. Skipped exon 10 cause the lack of KISc domain of KIF2C.

To know about whether the DAS events changed the corresponding protein structure, we analyzed the amino acid sequences and protein domains based on GenBank (https://www.ncbi.nlm.nih.gov/genbank/ accessed on 12 October 2021) and SMART databases (http://smart.embl-heidelberg.de/ accessed on 12 October 2021), and found two exon-skipping DASs that significantly changed protein structures. In the *exocyst complex component 7* (*EXOC7*) gene, the skipped exon8 deleted 23 amino acids and shortened the protein sequence, which frequency was significantly less in the MAP-infected group compared with the healthy cows (FDR_KS-test_ = 0.015) (Figure 4A). As for *kinesin family member 2C* (*KIF2C*) gene, there was a higher frequency (FDR_KS-test_ = 0.079) of exon 10 skipping event in the MAP-infected cows (Figure 4B), and it led to a stop codon of premature occurrence and shortened the protein sequence of 397 amino acids inducing the lack of KISc domain that plays important roles in intracellular transport of organelles and in cell division (Figure 4B).

## 4. Discussion

Paratuberculosis is an infectious disease that seriously endangers the health of dairy cows and causes huge economic losses to dairy farms. It is caused by MAP that invades macrophages and inhibits the immune response pathway. MAP invades the body and ushers along the gastrointestinal tract until it reaches the small intestinal mucosa where it enters microfold cells (M cells) and enterocytes to circumventing defense barriers of intestine such as the glycocalyx, tight junctions, and antimicrobial peptides. After that, MAP invades subcutaneous macrophages specifically to escape the host’s immune response and establish a long, chronic infection [43,44]. In the infection process, jejunum is the main affected tissue and manifests as inflammation and edema, inducing significant immune response such as activating interferon γ signaling [45]. In addition, some studies reported that MAP also invades the host from oral mucosa and be transported to small intestines through peripheral blood. Therefore, the salivary gland and peripheral blood are also important tissues of immune response in MAP infection, containing innate immune and adaptive immune [46,47]. It has been reported that salivary secretions such as defensins and cathelicidins have enormous significances for the regulation of the intestinal function and immune [48,49]. In this study, we integrated 6 jejunum RNA-seq datasets from our previous study [9] and 18 RNA-seq datasets from EMBL-EBI database, and identified 119 (90 genes), 150 (89 genes) and 68 (45 genes) DAS events between MAP-infected and healthy Holstein cows in peripheral blood, jejunum and salivary gland, respectively. Out of these, two DAS events in the *EXOC7* and *KIF2C* gene were associated with susceptibility and resistance to MAP.

EXOC7 is a component of the exocyst complex that plays a critical role in vesicular trafficking and the secretory pathway by targeting post-Golgi vesicles to the plasma membrane. The *EXOC7* gene consists of 21 exons that generates 9 types of known transcripts through different splicing events in cattle [50]. In a previous study, knockdown of the *EXOC7* gene inhibited the accumulation of vesicles at the phagosomes, leading to an inhibitory effect on bacterial elimination such as staphylococci and Salmonella [51]. Another research also showed that different transcripts of *EXOC7* influenced the efficiency of vesicle production and cytokines transport related to diabetes and inflammation [52]. Thus, the inhibited AS event of exon 8 of *EXOC7* in MAP-infected cows may affect immune response by reducing the efficiency of cytokines transport that is related to the susceptibility and resistance to diseases.

The *KIF2C* gene encodes a kinesin-like protein that functions as a microtubule-dependent molecular motor that depolymerizes microtubules at the plus end thereby promots mitotic chromosome segregation. It plays essential roles in the immune cells migration and infiltration in human [53]. Knockdown of *KIF2C* in mouse inhibited the apoptosis of CD8^+^ T cell and affected the immune cells infiltration that was associated with endometrial cancer [54]. In this study, the exon10-skipping event with higher frequency in MAP-infected cows might affect immune response to MAP-infection through inhibiting the function of immune cells.

**Figure 5:**
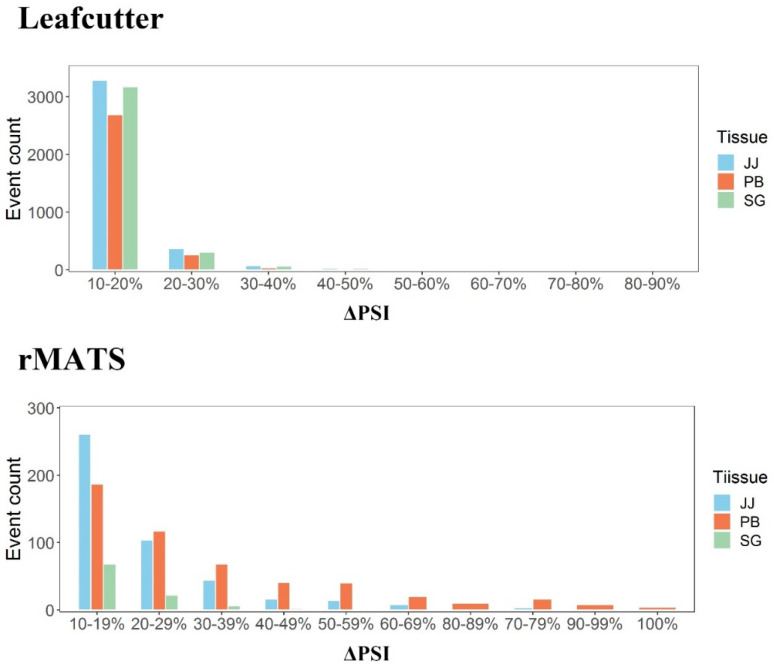
The distribution of DAS events in different regions of ΔPSI value. The figure above is the distribution calculated by leafcutter, and below is that by rmats. Of them, blue parts mean DAS events in jejunum, red parts mean that in peripheral blood and green parts mean that in salivary gland.

In this study, we performed two methods to identify the DAS events to increase accuracy of DASs [55]. Through the distribution of DAS events in different regions of ΔPSI value, we found that rMATS is more stringent in judging qualified DAS events, whereas there were less DAS events with high ΔPSI value (ΔPSI ≥ 70%) with leafcutter (Figure 5). On the other hand, merely 10% DAS events identified with both methods were used for analysis, and this may increase false negatives and discard some valuable information. Low overlap (10–30%) of results between different software was also reported in previous study which was caused by the false positives in AS identification [55]. In addition, more investigations are needed to verify the impacts of the DAS events in *EXOC7* and *KIF2C* on protein structures and functions and their regulatory mechanism underlying paratuberculosi in dairy cattle.

## 5. Conclusions

In the present study, based on 24 RNA-seq datasets from 3 tissues, we detected 224 genes that contained differential alternative splicing (DAS) events between MAP-infection and heathy Holstein cows, out of which 14 genes were significantly enriched for immune-related pathways. Of note, we found that the DAS events in the *EXOC7* and *KIF2C* changed protein structures and were associated with the susceptibility to MAP. Our findings provided novel insights into the regulatory mechanisms underlying paratuberculosis in dairy cattle.

## Figures and Tables

**Table 1:** Detailed information about the RNA-seq data.

Trait Material	Data Grouping	Sequencing Platform	Breed	Data Sources	Accession Numbers
Jejunum	NP, *n* = 3 DP, *n* = 3	Illumina Hiseq 2500 (Bos taurus)	Chinese Holstein cows	Gao Yahui, 2017	PRJNA756737
Peripheral blood	control, *n* = 2 case, *n* = 2	Illumina NovaSeq 6000 (Bos taurus)	Korean Holstein cows	Seoul National University, Park H, Yoo H, 2020	PRJNA628877
Salivary Gland	control, *n* = 7 case, *n* = 7	Illumina NovaSeq 6000 (Bos taurus)	Irish Holstein cows	University of Guelph, Mallikarjunappa S, 2019	PRJNA513864

## Data Availability

The datasets are available in the NCBI BioProject database under the accession number PRJNA756737, PRJNA628877 and PRJNA513864.

## Supplementary Materials

The following supporting information can be downloaded at: https://www.mdpi.com/article/10.3390/genes13030497/s1, Supplementary Table S1: Identified DAS events in two software, Supplementary Table S2: Results of correlation analysis between DESF and DAS.

## References

[1] Clarke C.J. (1997). The pathology and pathogenesis of paratuberculosis in ruminants and other species. J. Comp. Pathol..

[2] Stevenson K., Alvarez J., Bakker D., Biet F., de Juan L., Denham S., Dimareli Z., Dohmann K., Gerlach G.F., Heron I. (2009). Occurrence of Mycobacterium avium subspecies paratuberculosis across host species and European countries with evidence for transmission between wildlife and domestic ruminants. BMC Microbiol..

[3] Garcia A.B., Shalloo L. (2015). Invited review: The economic impact and control of paratuberculosis in cattle. J. Dairy Sci..

[4] Smith R.L., Strawderman R.L., Schukken Y.H., Wells S.J., Pradhan A.K., Espejo L.A., Whitlock R.H., Van Kessel J.S., Smith J.M., Wolfgang D.R. (2010). Effect of Johne’s disease status on reproduction and culling in dairy cattle. J. Dairy Sci..

[5] Gonda M.G., Kirkpatrick B.W., Shook G.E., Collins M.T. (2007). Identification of a QTL on BTA20 affecting susceptibility to Mycobacterium avium ssp. paratuberculosis infection in US Holsteins. Anim. Genet..

[6] Pant S.D., Schenkel F.S., Verschoor C.P., You Q., Kelton D.F., Moore S.S., Karrow N.A. (2010). A principal component regression based genome wide analysis approach reveals the presence of a novel QTL on BTA7 for MAP resistance in holstein cattle. Genomics.

[7] Kiser J.N., White S.N., Johnson K.A., Hoff J.L., Taylor J.F., Neibergs H.L. (2017). Identification of loci associated with susceptibility to subspecies tissue infection in cattle. J. Anim. Sci..

[8] Gao Y., Jiang J., Yang S., Cao J., Han B., Wang Y., Zhang Y., Yu Y., Zhang S., Zhang Q. (2018). Genome-wide association study of Mycobacterium avium subspecies Paratuberculosis infection in Chinese Holstein. BMC Genom..

[9] Gao Y. (2017). Identification of Susceptibility/Resistance and Milk Component Trait Functional Genes of Para-Tuberculosis in Dairy Cows Based on Omics Technique.

[10] Wilkinson M.E., Charenton C., Nagai K. (2020). RNA Splicing by the Spliceosome. Annu. Rev. Biochem..

[11] Chen M., Manley J.L. (2009). Mechanisms of alternative splicing regulation: Insights from molecular and genomics approaches. Nat. Rev. Mol. Cell Biol..

[12] Baralle F.E., Giudice J. (2017). Alternative splicing as a regulator of development and tissue identity. Nat. Rev. Mol. Cell Biol..

[13] Wang J., Zhang J., Li K., Zhao W., Cui Q. (2012). SpliceDisease database: Linking RNA splicing and disease. Nucleic Acids Res..

[14] Montes M., Sanford B.L., Comiskey D.F., Chandler D.S. (2019). RNA Splicing and Disease: Animal Models to Therapies. Trends Genet. TIG.

[15] Wobst H.J., Denk F., Oliver P.L., Livieratos A., Taylor T.N., Knudsen M.H., Bengoa-Vergniory N., Bannerman D., Wade-Martins R. (2017). Increased 4R tau expression and behavioural changes in a novel MAPT-N296H genomic mouse model of tauopathy. Sci. Rep..

[16] Qiu H., Lee S., Shang Y., Wang W.Y., Au K.F., Kamiya S., Barmada S.J., Finkbeiner S., Lui H., Carlton C.E. (2014). ALS-associated mutation FUS-R521C causes DNA damage and RNA splicing defects. J. Clin. Investig..

[17] Liu S.M., Sutherland A.P., Zhang Z., Rainbow D.B., Quintana F.J., Paterson A.M., Sharpe A.H., Oukka M., Wicker L.S., Kuchroo V.K. (2012). Overexpression of the Ctla-4 isoform lacking exons 2 and 3 causes autoimmunity. J. Immunol..

[18] Wang X.G., Ju Z.H., Hou M.H., Jiang Q., Yang C.H., Zhang Y., Sun Y., Li R.L., Wang C.F., Zhong J.F. (2016). Deciphering Transcriptome and Complex Alternative Splicing Transcripts in Mammary Gland Tissues from Cows Naturally Infected with Staphylococcus aureus Mastitis. PLoS ONE.

[19] Yang L., Guo R., Ju Z., Wang X., Jiang Q., Liu Y., Zhao H., He K., Li J., Huang J. (2019). Production of an aberrant splice variant of CCL5 is not caused by genetic mutation in the mammary glands of mastitis-infected Holstein cows. Mol. Med. Rep..

[20] Liang G., Malmuthuge N., Guan Y., Ren Y., Griebel P.J., le Guan L. (2016). Altered microRNA expression and pre-mRNA splicing events reveal new mechanisms associated with early stage Mycobacterium avium subspecies paratuberculosis infection. Sci. Rep..

[21] Emmert D.B., Stoehr P.J., Stoesser G., Cameron G.N. (1994). The European Bioinformatics Institute (EBI) databases. Nucleic Acids Res..

[22] Gao Y., Cao J., Zhang S., Zhang Q., Sun D. (2018). Short communication: Heritability estimates for susceptibility to Mycobacterium avium ssp. paratuberculosis infection in Chinese Holstein cattle. J. Dairy Sci..

[23] Alonso-Hearn M., Canive M., Blanco-Vazquez C., Torremocha R., Balseiro A., Amado J., Varela-Martinez E., Ramos R., Jugo B.M., Casais R. (2019). RNA-Seq analysis of ileocecal valve and peripheral blood from Holstein cattle infected with Mycobacterium avium subsp. paratuberculosis revealed dysregulation of the CXCL8/IL8 signaling pathway. Sci. Rep..

[24] Mallikarjunappa S., Adnane M., Cormican P., Karrow N.A., Meade K.G. (2019). Characterization of the bovine salivary gland transcriptome associated with Mycobacterium avium subsp. paratuberculosis experimental challenge. BMC Genom..

[25] Qi W., Schlapbach R., Rehrauer H. (2017). RNA-Seq Data Analysis from Raw Data Quality Control to Differential Expression Analysis. Methods Mol. Biol..

[26] Rosen B.D., Bickhart D.M., Schnabel R.D., Koren S., Elsik C.G., Tseng E., Rowan T.N., Low W.Y., Zimin A., Couldrey C. (2020). De novo assembly of the cattle reference genome with single-molecule sequencing. GigaScience.

[27] Howe K.L., Achuthan P., Allen J., Allen J., Alvarez-Jarreta J., Amode M.R., Armean I.M., Azov A.G., Bennett R., Bhai J. (2021). Ensembl 2021. Nucleic Acids Res..

[28] Dobin A., Davis C.A., Schlesinger F., Drenkow J., Zaleski C., Jha S., Batut P., Chaisson M., Gingeras T.R. (2013). STAR: Ultrafast universal RNA-seq aligner. Bioinformatics.

[29] Liao Y., Smyth G.K., Shi W. (2014). featureCounts: An efficient general purpose program for assigning sequence reads to genomic features. Bioinformatics.

[30] Love M.I., Huber W., Anders S. (2014). Moderated estimation of fold change and dispersion for RNA-seq data with DESeq2. Genome Biol..

[31] Zhou Y., Zhou B., Pache L., Chang M., Khodabakhshi A.H., Tanaseichuk O., Benner C., Chanda S.K. (2019). Metascape provides a biologist-oriented resource for the analysis of systems-level datasets. Nat. Commun..

[32] Yu G., Wang L.G., Han Y., He Q.Y. (2012). clusterProfiler: An R package for comparing biological themes among gene clusters. Omics.

[33] Shen S., Park J.W., Lu Z.X., Lin L., Henry M.D., Wu Y.N., Zhou Q., Xing Y. (2014). rMATS: Robust and flexible detection of differential alternative splicing from replicate RNA-Seq data. Proc. Natl. Acad. Sci. USA.

[34] Li Y.I., Knowles D.A., Humphrey J., Barbeira A.N., Dickinson S.P., Im H.K., Pritchard J.K. (2018). Annotation-free quantification of RNA splicing using LeafCutter. Nat. Genet..

[35] Jin Y.J., Byun S., Han S., Chamberlin J., Kim D., Kim M.J., Lee Y. (2019). Differential alternative splicing regulation among hepatocellular carcinoma with different risk factors. BMC Med. Genom..

[36] Du J.X., Zhu G.Q., Cai J.L., Wang B., Luo Y.H., Chen C., Cai C.Z., Zhang S.J., Zhou J., Fan J. (2021). Splicing factors: Insights into their regulatory network in alternative splicing in cancer. Cancer Lett..

[37] Giulietti M., Piva F., D’Antonio M., D’Onorio De Meo P., Paoletti D., Castrignanò T., D’Erchia A.M., Picardi E., Zambelli F., Principato G. (2013). SpliceAid-F: A database of human splicing factors and their RNA-binding sites. Nucleic Acids Res..

[38] Shannon P., Markiel A., Ozier O., Baliga N.S., Wang J.T., Ramage D., Amin N., Schwikowski B., Ideker T. (2003). Cytoscape: A software environment for integrated models of biomolecular interaction networks. Genome Res..

[39] Smoot M.E., Ono K., Ruscheinski J., Wang P.L., Ideker T. (2011). Cytoscape 2.8: New features for data integration and network visualization. Bioinformatics.

[40] Massey F.J. (1951). The Kolmogorov-Smirnov test for goodness of fit. J. Am. Stat. Assoc..

[41] (2018). NCBI Resource Coordinators: Database resources of the National Center for Biotechnology Information. Nucleic Acids Res..

[42] Schultz J., Copley R.R., Doerks T., Ponting C.P., Bork P. (2000). SMART: A web-based tool for the study of genetically mobile domains. Nucleic Acids Res..

[43] Bannantine J.P., Bermudez L.E. (2013). No holes barred: Invasion of the intestinal mucosa by Mycobacterium avium subsp. paratuberculosis. Infect. Immun..

[44] Arsenault R.J., Maattanen P., Daigle J., Potter A., Griebel P., Napper S. (2014). From mouth to macrophage: Mechanisms of innate immune subversion by Mycobacterium avium subsp. paratuberculosis. Vet. Res..

[45] Määttänen P., Trost B., Scruten E., Potter A., Kusalik A., Griebel P., Napper S. (2013). Divergent immune responses to Mycobacterium avium subsp. paratuberculosis infection correlate with kinome responses at the site of intestinal infection. Infect. Immun..

[46] Payne J.M., Rankin J.D. (1961). The Pathogenesis of Experimental Johne’s Disease in Calves. Res. Vet. Sci..

[47] Sweeney R.W., Uzonna J., Whitlock R.H., Habecker P.L., Chilton P., Scott P. (2006). Tissue predilection sites and effect of dose on Mycobacterium avium subs. paratuberculosis organism recovery in a short-term bovine experimental oral infection model. Res. Vet. Sci..

[48] Fábián T.K., Hermann P., Beck A., Fejérdy P., Fábián G. (2012). Salivary defense proteins: Their network and role in innate and acquired oral immunity. Int. J. Mol. Sci..

[49] Mathews M., Jia H.P., Guthmiller J.M., Losh G., Graham S., Johnson G.K., Tack B.F., McCray P.B. (1999). Production of β-defensin antimicrobial peptides by the oral mucosa and salivary glands. Infect. Immun..

[50] Zimin A.V., Delcher A.L., Florea L., Kelley D.R., Schatz M.C., Puiu D., Hanrahan F., Pertea G., Van Tassell C.P., Sonstegard T.S. (2009). A whole-genome assembly of the domestic cow, Bos taurus. Genome Biol..

[51] Wang S., Crisman L., Miller J., Datta I., Gulbranson D.R., Tian Y., Yin Q., Yu H., Shen J. (2019). Inducible Exoc7/Exo70 knockout reveals a critical role of the exocyst in insulin-regulated GLUT4 exocytosis. J. Biol. Chem..

[52] Georgilis A., Klotz S., Hanley C.J., Herranz N., Weirich B., Morancho B., Leote A.C., D’Artista L., Gallage S., Seehawer M. (2018). PTBP1-Mediated Alternative Splicing Regulates the Inflammatory Secretome and the Pro-tumorigenic Effects of Senescent Cells. Cancer Cell.

[53] Wang D., Liu J., Liu S., Li W. (2020). Identification of Crucial Genes Associated With Immune Cell Infiltration in Hepatocellular Carcinoma by Weighted Gene Co-expression Network Analysis. Front. Genet..

[54] An L., Zhang J., Feng D., Zhao Y., Ouyang W., Shi R., Zhou X., Yu Z., Wei S., Min J. (2021). KIF2C Is a Novel Prognostic Biomarker and Correlated with Immune Infiltration in Endometrial Cancer. Stem Cells Int..

[55] Mehmood A., Laiho A., Venäläinen M.S., McGlinchey A.J., Wang N., Elo L.L. (2020). Systematic evaluation of differential splicing tools for RNA-seq studies. Brief. Bioinform..

